# The cost of indirect billing for traditional Medicare beneficiaries

**DOI:** 10.1093/haschl/qxaf248

**Published:** 2025-12-24

**Authors:** Hannah T Neprash, John F Mulcahy

**Affiliations:** Division of Health Policy and Management, University of Minnesota School of Public Health, Minneapolis, MN 55455, United States; Department of Health Policy and Management, Johns Hopkins Bloomberg School of Public Health, Baltimore, MD 21205, United States

**Keywords:** Medicare payment policy, nurse practitioners, clinical workforce, patient out-of-pocket costs

Advanced practice clinicians (APCs) provide a growing share of services for Medicare beneficiaries.^1^ When caring for Medicare beneficiaries, APCs can bill Medicare directly and receive 85% of the Physician Fee Schedule rate for a service, or they can bill indirectly (ie, “incident to” the services of a physician) and receive 100% of the fee schedule rate. Indirect billing is costly to the Medicare program and existing research quantifies this.^2^ However, indirect billing also imposes a cost on Medicare beneficiaries, who frequently face cost-sharing based on Medicare reimbursement—and no empirical estimates exist to quantify this cost to beneficiaries. We analyzed traditional Medicare claims data to quantify the annual cost-sharing liability for Medicare beneficiaries and quantify characteristics of beneficiaries facing exposure to indirect billing and higher cost-sharing liabilities as result.

## Methods

Using 100% of fee-for-service claims, we applied existing methods to identify office-based Physician Fee Schedule services provided by APCs and billed indirectly to Medicare.^1-3^ In brief, these methods link prescription drug claims to claims for office services and rely on prescriber identifiers to identify indirect billing by an APC, before extrapolating patterns of indirect billing to non-prescription-linked encounters, in order to estimate overall prevalence and spending. We then calculated the total annual cost-sharing liability (the sum of deductible and coinsurance liabilities) for care provided by APCs and billed directly or indirectly. To identify potential savings from the elimination of indirect billing, we calculated 15% of all indirectly-billed cost-sharing liability.

To test for beneficiary characteristics associated with exposure to any indirect billing and with a higher financial burden of indirect billing, we focused on beneficiaries with full-year enrollment in traditional Medicare who received any care from an APC in 2023. We first regressed an indicator for whether any APC-provided care was billed indirectly on beneficiary characteristics. Beneficiary characteristics of interest included age category, sex, race, dual eligibility for Medicare and Medicaid, rural location, and chronic condition count. We next focused on beneficiaries with >$0 cost-sharing liability for indirectly-billed services provided by APCs in 2023. We regressed the log of beneficiary-level total cost-sharing liability for indirectly-billed services on beneficiary characteristics. Taking the log of a skewed spending variable such as cost-sharing liability is standard practice for skewed spending variables and results in an interpretation of coefficient as percent change. Both dependent variables relied solely on our subsample of prescription-linked encounters where we can observe indirect-billing directly, as our imputation method is imprecise at the beneficiary-level. The University of Minnesota institutional review board approved analyses, which were conducted using SAS version 9.4.

## Results

**Figure 1.**
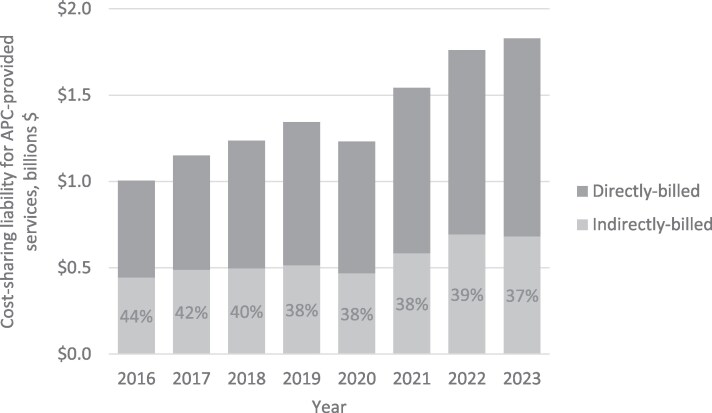
Traditional medicare beneficiary cost-sharing liability for advanced practice clinician (APC)-provided office-based services by billing Status from 2016 to 2023. Figure displays total annual cost-sharing liability for office-based Physician Fee Schedule services delivered by APCs to FFS Medicare beneficiaries, distinguishing directly- and indirectly-billed services. APC is advanced practice clinician.

Our sample included 50 843 425 beneficiaries enrolled in traditional Medicare between 2016 and 2023. Figure 1 shows annual cost-sharing liability for Physician Fee Schedule services provided by APCs, by billing status (ie, direct and indirectly-billed services). In 2023, traditional Medicare beneficiaries accrued a total of $682.9 million in cost-sharing liability for indirectly-billed APC-provided services. Had these same services been provided by APCs and billed directly to Medicare, cost-sharing liability would have been $102.4 million lower.

Table 1 show that some beneficiary characteristics were associated with exposure to indirect billing and with a higher financial burden of indirect billing. Among beneficiaries who received care from APCs in 2023, predictors associated with exposure to indirect billing included dual eligibility for Medicare and Medicaid (5.94pp; 95% CI 5.81-6.06pp), Medicare eligibility due to disability (3.24pp; 95% CI 3.10-3.37pp), self-reported race/ethnicity of Asian/Pacific Islander (7.24pp; 95% CI 6.95-7.53pp), Hispanic (7.26pp; 95% CI 7.08-7.45pp), or Black (2.03pp; 95% CI 1.86-2.20pp), and having two, three, or four plus chronic conditions. Among beneficiaries who had at least one indirectly-billed service in 2023, predictors associated with higher annual cost-sharing liability on indirectly-billed APC-provided office-based care included younger age (among age-eligible beneficiaries), dual eligibility for Medicare and Medicaid (7.2%; 95% CI 6.9%-7.5%), Medicare eligibility due to disability (4.4%; 95% CI 4.1%-4.8%), and self-reported race/ethnicity of Asian/Pacific Islander (12.5%; 95% CI 11.8%-13.2%) or Hispanic (12.7%; 95% CI 12.2%-13.1%).

**Table 1.** Beneficiary predictors of cost-sharing liability for indirectly-billed APC-provided office-based services in 2023.

Characteristic	Percentage point change in likelihood of any indirectly-billed services (95% CI)	Percent change in cost-sharing for indirectly-billed services (95% CI)
Female	−2.00 (−2.08 to −1.92)	0.02 (0.02-0.02)
Age		
Under 65	−5.69 (−5.87 to −5.50)	−0.01 (−0.01 to −0.004)
65-69	[Reference]	[Reference]
70-74	−0.97 (−1.09 to −0.85)	−0.02 (−0.02 to −0.02)
75-79	0.21 (0.08-0.34)	−0.03 (−0.04 to −0.03)
80-84	0.82 (0.67-0.97)	−0.05 (−0.06 to −0.05)
85+	0.84 (0.69-1.00)	−0.08 (−0.09 to −0.08)
Dual-eligible	5.94 (5.81-6.06)	0.07 (0.07-0.07)
Disability	3.24 (3.10-3.37)	0.04 (0.04-0.04)
Race/ethnicity		
American Indian/Alaska Native	− 8.93 (−9.61 to −8.26)	−0.08 (−0.09 to −0.08)
Asian/Pacific Islander	7.24 (6.95-7.53)	0.13 (0.12-0.13)
Non-Hispanic Black	2.03 (1.86-2.20)	0.004 (−0.001-0.01)
Hispanic	7.26 (7.08-7.45)	0.13 (0.12-0.13)
Other	1.90 (1.36-2.43)	0.04 (0.02-0.05)
Unknown	0.67 (0.41-0.93)	−0.01 (−0.02 to −0.002)
White	[Reference]	[Reference]
Rural	−5.94 (−6.03 to −5.84)	−0.1 (−0.11 to −0.1)
Chronic condition count		
0	[Reference]	[Reference]
1	−0.66 (−0.80 to −0.53)	−0.03 (−0.03 to −0.02)
2	3.98 (3.85-4.12)	−0.05 (−0.05 to −0.04)
3	9.30 (9.17-9.44)	−0.04 (−0.05 to −0.04)
4+	19.07 (18.95-19.19)	0.01 (0.01-0.02)

Table displays coefficients from a multivariate regression of exposure to any indirectly-billed services in 2023 on beneficiary characteristics (column 2) and log spending on indirectly-billed cost-sharing for office-based services in 2023 on beneficiary characteristics (column 3).

## Discussion

Indirect billing by APCs is not only costly to the taxpayer-funded Medicare program, but also to Medicare beneficiaries. In 2023, the elimination of indirect billing would have saved traditional Medicare beneficiaries more than $100 million. The cost-sharing liability of indirect billing falls more heavily on patient populations that may already struggle with the cost of care, including beneficiaries who are dually eligible for Medicare and Medicaid, beneficiaries whose Medicare eligibility is due to disability, and Medicare beneficiaries of color. While this finding reflects many complex factors that determine who receives how many services and from which clinicians, it also suggests that Medicare's current payment policies for APC-provided care result in a higher financial burden for potentially vulnerable patient populations with traditional Medicare coverage.

Limitations of this work include a focus solely on traditional Medicare beneficiaries. While past work has documented similar quantities of indirect billing in Medicare Advantage,^2^ encounter data do not include cost-sharing or other payment amounts and therefore cannot be included in these analyses. Additionally, our measure of cost-sharing liability may not reflect beneficiaries’ true out-of-pocket obligation, among the majority of those who have supplemental coverage (eg, Medigap coverage). The fact that supplemental coverage rates are lower among dual-eligible and disabled Medicare beneficiaries likely means that estimates in this paper downplay differences in true out-of-pocket obligation resulting from indirect billing.^4^

## Supplementary Material

qxaf248_Supplementary_Data
